# The Effects of Absorbing Materials on the Homogeneity of Composite Heating by Microwave Radiation

**DOI:** 10.3390/ma14237362

**Published:** 2021-11-30

**Authors:** Betime Nuhiji, Matthew P. Bower, William A. E. Proud, Steven J. Burpo, Richard J. Day, Richard J. Scaife, Timothy Swait

**Affiliations:** 1 Advanced Manufacturing Research Centre, University of Sheffield, Wallis Way, Catcliffe, Rotherham S60 5TZ, UK; mbower15@googlemail.com (M.P.B.); william.ae.proud@gmail.com (W.A.E.P.); richard.scaife@sheffield.ac.uk (R.J.S.); t.swait@sheffield.ac.uk (T.S.); 2 The Boeing Company, St. Louis, MO 63134, USA; steven.j.burpo@boeing.com; 3 Faculty of Arts, Science and Technology, Wrexham Glyndŵr University, Plas Coch, Mold Road, Wrexham LL11 2AW, UK; r.day@glyndwr.ac.uk

**Keywords:** polymer-matrix composites (PMCs), carbon fibre, cure behaviour, microwave absorbing materials

## Abstract

When cured in a microwave, flat thin composite panels can experience even heat distribution throughout the laminate. However, as load and geometric complexity increase, the electromagnetic field and resulting heat distribution is altered, making it difficult to cure the composite homogeneously. Materials that absorb and/or reflect incident electromagnetic radiation have the potential to influence how the field behaves, and therefore to tailor and improve the uniformity of heat distribution. In this study, an absorber was applied to a composite with non-uniform geometry to increase heating in the location which had previously been the coldest position, transforming it into the hottest. Although this result overshot the desired outcome of temperature uniformity, it shows the potential of absorbing materials to radically change the temperature distribution, demonstrating that with better regulation of the absorbing effect, a uniform temperature distribution is possible even in non-uniform composite geometries.

## 1. Introduction

High rate curing technologies are increasingly being investigated to manufacture composites [1,2,3,4,5], given the demand for these high strength, low weight materials within industry, such as for aerospace and automotive application. Of these technologies, microwaves can offer significant benefits, such as selective and volumetric heating of composite materials, high heating rates (reduction of processing time), and energy efficiency [6]. To obtain these advantages on an industrial scale, without a reduction in composite performance, the manufacturing method must be optimised accordingly. This requires an appreciation of how materials behave when exposed to an electromagnetic (EM) environment [7,8,9,10], including tools and composite parts [11,12,13], as well as the use of absorbers (also referred to as susceptors) [14,15,16,17].

Materials can be transparent, absorbing, or reflective (or a combination) to microwave irradiation. The complex permittivity (*ԑ_r_* = *ԑ*′ + 𝑖*ԑ*”) is an important parameter as it describes the behaviour of a material in an EM field, mainly in terms of its absorption properties. Absorber materials are traditionally used to eliminate EM wave pollution and reduce signs of radar for civil and military applications [14,18,19]. These materials can originate in the form of powders as well as binders and, as the name suggests, can absorb EM energy due to their high dielectric characteristics. By absorbing a greater amount of the incident radiation, microwave absorbing materials (MAMs) can also be used for shielding electronic equipment and in stealth technology for defence [14,15,20]. With these materials demonstrating the ability to absorb EM energy and in turn manipulate the field, they also have the potential to be applied in a different manner to facilitate in the curing of composite materials. For complex geometries consisting of varying thicknesses, high loss MAMs can be explored to selectively couple more readily with the microwaves and in turn generate a more homogenous temperature distribution across and throughout the laminate.

To understand how a material behaves when exposed to an EM field, information such as frequency, fillers, and operating temperatures need to be taken into consideration. Radar applications utilise radar absorbing materials (RAMs) to focus an EM field on objects for industrial use. These materials operate at a high frequency, such as the X-band range of 8 GHz–12 GHz, and may consist of materials including silicon with a conductive polymer (polyaniline) [21], carbon fibre with polyaniline [22], and carbon black and/or with glass/epoxy composites [23,24]. Although the behaviour of these materials is frequency dependant and they have the potential to behave differently at 2.45 GHz (industrial, scientific, and medical (ISM) standard), they show promise towards industrial scale-up as the selection is based upon targeting lightweight, thin, and flexible materials. Additionally, the fillers used are conductors, which are similar to those required to absorb EM energy at 2.45 GHz. In terms of operating temperatures, a material’s dielectric properties can alter during a cure cycle, as it undergoes physical and structural transformations [25]. For thermosetting polymers, the molecular network will crosslink when heated; however, this could also occur in other materials associated with the composite cure, such as MAMs. The behaviour of materials when exposed to EM energy has been highlighted as a challenging area, as dielectric properties can change as a function of time and temperature [26]. Therefore, it is important to measure the dielectric response of a MAM at elevated temperatures, or even when simulating a cure cycle. Understanding these properties will allow researchers to design and implement the materials and processes accordingly.

This study investigates the influence of MAMs when applied to a carbon fibre composite geometry consisting of a variable thickness, in order to enhance heating in regions that are otherwise not sufficiently heated. Bespoke and commercial absorbers were tested for their dielectric and thermal properties, and these materials (alongside composites) were trialled on laboratory and industrial scales, with the aim to determine whether an absorber can be used to attract the EM field and, in turn, enhance the heat distribution in a composite part.

## 2. Materials and Methods

### 2.1. Materials, Equipment, and Manufacturing

**Figure 1:**
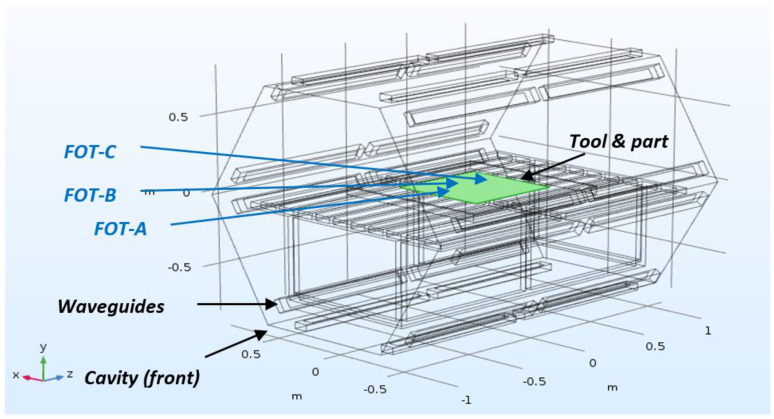
Schematic illustration of the Vötsch microwave with the tool and part in the centre of the cavity [13].

A test bed and an industrial Vötsch HEPHAISTOS VHM 180/200 microwave system operating at a frequency of 2.45 GHz were used, where temperature monitoring and control were conducted using fibre optic thermosensors (FOTs) with an accuracy to ±1 °C. The test bed is based on a single magnetron (1 kW) Panasonic NN-CF778 system, and uses a bespoke control designed to cure organic carbon fibre reinforced epoxy composite materials [7]. The Vötsch system has 24 magnetrons (21 kW) positioned in a hexagonal configuration designed to improve field homogeneity [6]. Two infrared (IR) cameras are located inside the roof of the Vötsch chamber to view the heat distribution of the composite (Figure 1). It should be noted that while the FOTs provide accurate temperature measurements at discrete points, the IR images give an indication of the spatial distribution of heat. However, due to consumables and bagging on the surface of the panel, these images cannot be used to obtain meaningful absolute temperatures of the composite.

**Figure 2:**
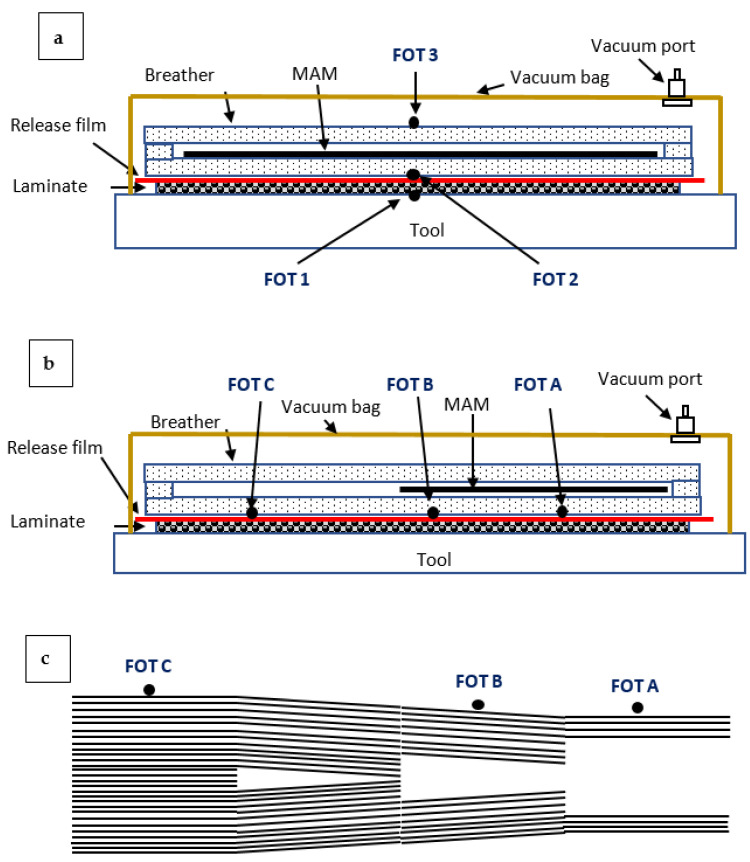
Schematic of the composite layup within the (**a**) test bed and (**b**) industrial microwaves (side of the microwave view). These diagrams specify the locations of the FOTs. A representation of the step geometry is shown in (**c**), where the internal ply drop for the panels can be seen.

Test bed trials were conducted on laminates processed using Cycom 5320-1 T650 3K plain weave prepreg. The panels consisted of 8 plies [0/90]_s_ of 150 mm × 150 mm prepreg that were shielded around the edges with aluminium tape to avoid potential arcing, and bagged using breather, sealant tape, release film, and vacuum bag. All panels were cured on a ceramic tool using the manufacturer’s recommended cure profile (121 °C for 180 min followed by 177 °C for 120 min with 3 °C/min ramp rates). As is standard practise for high performance fibre reinforced composites, a vacuum was applied to consolidate the panels. The FOTs were located in the centre of the CFRP panels at various layers of the laminate, where FOT-1 was located between the tool and the laminate, FOT-2 was between the laminate and the MAM, and FOT-3 was on top of the MAM (refer to Figure 2a). The power used throughout the curing process was monitored by the control system. The process was similar for industrial scale trials, although the CFRP laminates consisted of a 1000 mm × 1000 mm panel of stepped thickness, ranging from 10 mm to 2.5 mm thick in 2.5 mm increments. The thickest region of the panels was 32 plies, with each step dropping eight plies (Figure 2c). Figure 2c shows the ply schematic with the internal ply drops for the laminate. This layup was selected as the ply edges (which have the potential to arc and thermally degrade the resin if exposed) have been positioned internally.

Both bespoke and commercial MAMs were processed and used in this study to provide a wide range of dielectric properties. To manufacture the bespoke MAM (MAM-1), a two-part silicone (Silcoset 101) was combined with 35 wt% silicon carbide (SiC) and 5 wt% carbon black (CB), and distributed within the matrix using an Ultramix Silverston L4R high-shear mixer. These fillers were selected as they have been reported to exhibit excellent absorption properties when combined, and over a large frequency range [27]. An acrylonitrile butadiene styrene (ABS) mould was 3D printed (200 mm × 200 mm) to form MAM-1 into sheets. Once the MAM was processed, it was outgassed in a vacuum oven for 60 min before curing at room temperature for 24 h. A commercial rubber compound (Material SFL8925/26) containing aluminium and carbon black fillers was supplied by Kraiburg TPE GmbH & Co. KG and used as another MAM throughout this study (MAM-2). All MAM materials were located within the composite bag on top of the composite laminate amongst the breather material (Figure 2).

### 2.2. Characterisation

Dielectric and thermal diffusivity properties were measured and used to evaluate the behaviour of the MAMs, while differential scanning calorimetry (DSC) measured the composite’s degree of cure (DOC).

#### 2.2.1. Dielectric Properties

The dielectric properties of the absorbers were measured using the equipment outlined in previous work by the authors [13]. The two methods used include resonance cavity perturbation to measure non-laminar materials and a mode cavity method (Split-Post Dielectric Resonator (SPDR)) for laminar based materials. SPDR measurements were generated using Quickwave equipment and software in combination with an Anritsu Scorpion vector network analyser. In previous work, cavity perturbation measurements were made using a microwave calorimeter developed by Nesbitt et al. [28], with the addition of an amplifier (Microwave Amplifiers Ltd. Bristol, United Kingdom) and a network analyser (HP 8720 ET), at a single temperature. However, in this study, the measurements were conducted across an elevated temperature range using the cavity perturbation technique, with the temperature profile simulating a cure cycle, using the microwave calorimeter in order to assess the microwave absorption characteristics over the temperature range they were subject to (121 °C for 60 min with an additional heating increase to 177 °C at 3 °C/min).

#### 2.2.2. Thermal Diffusivity

The thermal diffusivity determines how a material responds to a change in temperature, and is the thermal conductivity divided by the volumetric specific heat capacity (Equation (1)).

(1) $$\alpha =\frac{k}{\rho C_{\rho }}$$

Here, *α* is the thermal diffusivity, *k* is conductivity (W/(m·K)), *ρ* is the density (kg/m³), and *C_ρ_* is the specific heat capacity (J/(kg·K)) of the MAMs. Volumetric specific heat can be stated as the density and specific heat of a material (*ρC_ρ_*). The diffusivity of the MAMs was measured using a TPS2500S HotDisk, and the results were interpreted with the Thermal Constants Analyser 7.2.6 software.

#### 2.2.3. Differential Scanning Calorimetry (DSC)

The heat capacity of the Cycom 5320-1 prepreg and any residual cure of the CFRP panels was measured using a Perkin Elmer DSC 4000. Samples were heated to 300 °C at 10 °C/min and cooled to room temperature, as per the standard protocol according to ASTM E1269–11. Once conducted, the CFRP parts were analysed for their DOC using Equation (2).

(2) $$DoC=\frac{\delta H\left(r\right)-\delta H\left(T\right)}{\delta H\left(T\right)}\times 100\%$$

Here, *δH*(*T*) is the total heat of reaction (J/g) and *δH*(*r*) is the residual heat of reaction (J/g).

## 3. Results and Discussion

### 3.1. Selection of an Effective Microwave Absorber Material (MAM)

**Figure 3:**
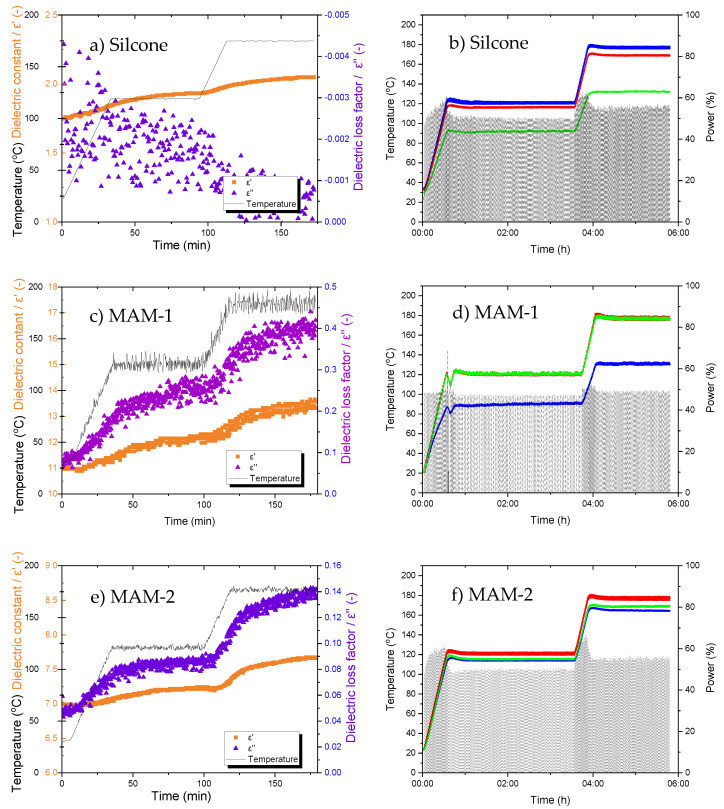
Dielectric properties and temperature-time-power graphs of (**a**,**b**) silicone, (**c**,**d**) MAM-1, and (**e**,**f**) MAM-2, respectively. The temperature-time-power graphs include the setpoint temperature (black solid), FOT-1 (blue), FOT-2 (red), FOT-3 (green), and power (black dash), where trials were conducted on the microwave test bed.

Two MAMs (bespoke and commercial) were tested for their dielectric and thermal diffusivity properties, alongside their effectiveness to influence the heat distribution of a composite in a microwave environment. Figure 3 displays the results of the dielectric properties as well as the temperature-time-power graphs generated from the microwave test bed, whereas Table 1 outlines the diffusivity and conductivity properties of the materials. The dielectric permittivity is a measure of stored energy, whereas the loss controls the heat that a material can generate [13]. Combined, the results facilitate the selection of a MAM for large-scale microwave manufacturing.

#### 3.1.1. Silicone Material (Matrix)

MAMs consist of a bulk material (known as a matrix) and a number of discrete filler materials that are highly absorbing. Whilst literature has generally used epoxide resins as the matrix material for MAMs [27], this study requires the material to have a high degree of formability for complex geometries.

Silicone rubber is commonly used for industrial applications as a matrix material due to its thermal properties (withstands temperatures above 250 °C), excellent reflectivity performance, and its ability to be formed into complex geometries enabling the MAM to conform to composite tooling when required [29]. Additionally, it has been reported that at 2.45 GHz, silicone has a low dielectric loss factor, indicating that microwaves can penetrate into the material freely and will not be reflected at the surface [29]. It is for these reasons that silicone was trialled as the matrix material to manufacture a bespoke MAM.

From the dielectric data in Figure 3, the silicone’s constant (ε’) and loss factor (ε’’) did alter with temperature, although only slightly (Figure 3a). The material’s ε’ ranges from approximately 1.75 to 2, which implies it will facilitate the absorption of microwaves, although ε’’ remains at zero, indicating it will not readily heat in a microwave EM field during a cure cycle [30]. The temperature-time-power graph for the silicone can be seen in Figure 3b. From this graph, FOT-1 follows the temperature setpoint, whereas FOT-2 remains approximately 10 °C lower throughout the cure cycle. FOT-3, however, exhibits a temperature gradient of 30–50 °C. Given the locations of these FOTs, the composite laminate and the tool remain warmer than the silicone (FOT-3), showing that while the laminate is directly heated by the microwave field, the silicone is not, or is only slightly, heated. It is for this reason that the FOTs in contact with the laminate (1 and 2) are reading higher, while the FOT closest to the silicone (but without direct contact with the laminate) is significantly cooler. These results, in addition to the robustness of the material, suggest that silicone, while not able to enhance microwave heating alone, is a suitable matrix to combine with fillers to act as an absorber for EM processing. For this work, the silicone can also be thought of as a control when compared to the two highly absorbing materials.

#### 3.1.2. Bespoke Absorber (MAM-1)

The dielectric properties of MAM-1 are shown in Figure 3c. When silicone is blended with SiC and CB, the material’s dielectric properties increase with temperature. The ε’’ ranges from 0.1 to 0.4 (increase in loss by a factor of 4 when measured at room temperature) and the ε’ increases from 11 to 13. These results are positive, as the fillers have enhanced the silicone’s microwave properties such that MAM-1 is absorbing the EM field and heating the composite laminate. When comparing the dielectric results (Figure 3c) to the temperature-time-power graph (Figure 3d), both the FOTs associated with the absorber (2 and 3) are following the cure setpoint temperature closely, therefore supporting the results in Figure 3c. However, the FOT on the tool face of the laminate (FOT-1) is not effectively heated, lagging by approximately 30 °C. In this case, it appears that the absorber is too effective; it is self-heating very strongly, but in doing so is preventing effective heating of the actual laminate.

#### 3.1.3. Commercial Absorber (MAM-2)

The results for the dielectric properties of MAM-2 are shown in Figure 3e and are accompanied by the temperature-time-power graph in Figure 3f. Similar to the dielectric properties of MAM-1, the ε’ and ε’’ of MAM-2 also increase with temperature. The ε’ ranges from 7 to 7.5 and the ε’’ from 0.05 to 0.14 (ε’’ increases by a factor of 2 when measured at room temperature), indicating that these qualities are less pronounced than the dielectric properties of MAM-1, although still suggesting a strong absorbing effect compared to the unmodified silicone control. When combining this information with the results in Figure 3f, the temperature at all FOT locations remained within 20 °C throughout the heating profile, which is less than the temperature gradient experienced by MAM-1. From a manufacturing perspective, these results show that the composite panel exhibited greater temperature homogeneity of the composite when utilising MAM-2 during the curing process, and crucially the MAM did not appear to be significantly inhibiting the heating of the laminate while heating itself.

In addition to the dielectric and cure profile data, thermal diffusivity properties are also important when selecting a MAM, as the results indicate how quickly a material can transfer thermal energy. One of the well-known limitations of microwave curing is that of localised heating (hot spots) generated by standing waves [25,26]. This localised heating will lead to an inhomogeneous heat distribution, which causes variation in the state of cure and residual stresses in the component. Therefore, when using a MAM with a high diffusivity property, the temperature is distributed across the part more effectively, leading to a more homogeneous rate and state of cure in a flat composite panel.

Table 1 displays the thermal diffusivity and conductivity properties of silicone, MAM-1, and MAM-2. From this table, it is clear that both properties increase when fillers are used. The modified silicone based MAM (MAM-1) displayed increased thermal conductivity and diffusivity due to the addition of SiC and CB, which has also been evidenced by Liu et al. [27]. Silicone has a conductivity of 0.36 W/mK and a diffusivity of 0.21 mm/s^2^, whereas the addition of SiC and CB increased both these properties two-fold to 0.71 W/mK and 0.46 mm/s^2^, respectively. The thermal properties of the commercial MAM (MAM-2) were higher than those registered with MAM-1, with the diffusivity and conductivity measured as 0.90 W/mK and 1.39 mm/s^2^, respectively. This indicates that MAM-2 would be expected to homogenise the heat distribution across the composite panel in a microwave environment. Additionally, the diffusivity results support the temperature-time-power data for the composite cured with MAM-1 (Figure 3d), where the tool remained cooler than the MAM and composite, as the diffusivity was lower than that of MAM-2. The combined results in Section 3.1 prompted MAM-2 to be selected as the material for industrial scale trials with a complex composite geometry.

### 3.2. Industrial Composite Manufacturing

Prior to utilising MAM-2 for large-scale manufacturing, a composite laminate without a MAM was trialled in an industrial microwave to identify the locations of EM standing waves and consequent hotspots during the cure process. A second composite laminate was then manufactured using a MAM to control the homogeneity of the heating in the composite.

The laminates were located in the centre of the microwave cavity, where the locations of the FOTs relative to the cavity are shown in Figure 1. A stepped (varying in thickness from 2.5 to 10 mm) geometry was selected to evaluate the distribution of the EM radiation within the cavity over a part with varying dielectric load. Temperature readings were taken across the panel to establish which region heated most strongly. The microwave power was controlled by a PID control loop set to control from the highest reading FOT.

#### 3.2.1. Composite Laminate Manufactured without a MAM

**Figure 4:**
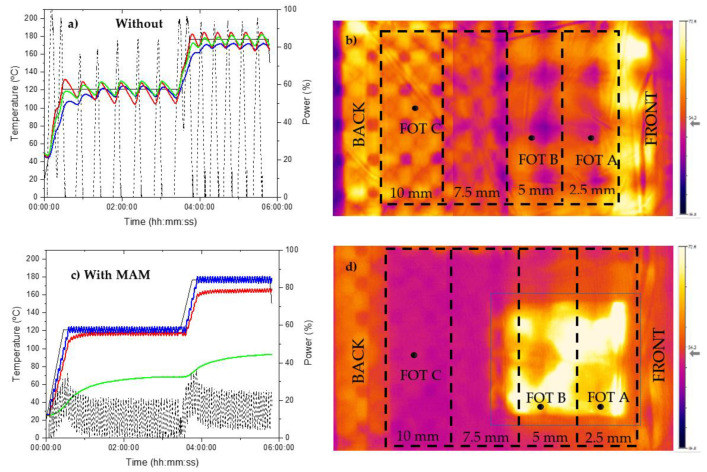
Results for the industrial microwave trials, including (**a**,**c**) cure cycle of temperature (black solid), FOT-A (blue), FOT-B (red), FOT-C (green), and power (black dash) for the composite cured without and with MAM-2, respectively, and (**b**,**d**) infrared images of the cures where the black line outlines the composite component and the dashed blue line represents the location of the MAM.

The temperature-time-power graph of the composite manufactured without a MAM is shown in Figure 4a and is accompanied with an IR image in Figure 4b. The image is taken from the two IR cameras positioned on the ceiling of the Vötsch cavity, where the front door of the microwave is located on the right of the image and the back of the cavity is to the left. The thicknesses of the laminates are listed on the image with FOT-A, FOT-B, and FOT-C measuring the temperatures of the composite at the 2.5 mm, 5 mm, and 10 mm regions, respectively.

The data in Figure 4a indicate that the temperature fluctuates throughout the cure cycle, regardless of the composite location being measured via the thermosensors. This shows that PID is not an ideal control strategy for this application. Better tuning of the PID parameters would provide an improved response, but this tuning would be highly dependent on the particular component that was being cured and could never be universal to differing components. Additionally, given that the response of the magnetrons and the resulting heating response of the material are highly non-linear, asymmetric with regard to heating and cooling, vary with temperature, and have significant lag, all of which are factors known to be problematic to a PID controller, it appears that a more sophisticated control strategy than PID is required. FOT-B and FOT-C readings follow a similar pattern along the set temperature and are located away from the front of the microwave, whereas FOT-A continuously remains below the set temperature with an offset of approximately 10 °C. From these results, it is evident that the middle and back of the panel (5 mm and 10 mm thickness, respectively) remain at the highest temperature throughout the cycle, whereas the front is the coldest (2.5 mm thickness). This was confirmed by thermal images taken throughout the cure cycle, such as those in Figure 4. This could be due to a combination of factors contributing to this effect, such as a greater dielectric load in the thicker regions, enabling radiation to be absorbed more readily, as well as the extra energy produced by a large exothermic reaction, as more resin is in this region while the thicker laminate results in a higher volume to surface area ratio and thus lower thermal losses.

**Figure 5:**
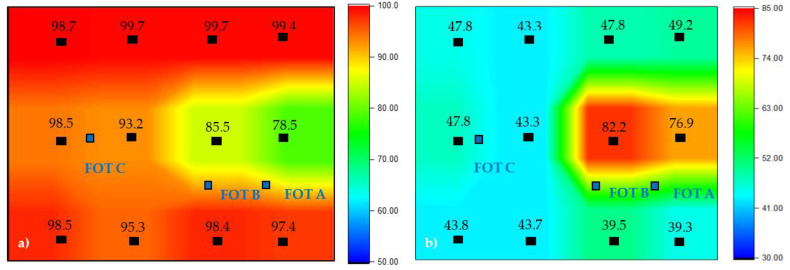
False colour of image of the degree of cure across the microwave cured panel (**a**) without a MAM and (**b**) with MAM-2. The points indicated are the locations where DSC samples were measured and their respective cured percentage values.

To confirm that the composite panel was cured across its area, DSC was conducted. The enthalpy of reaction of as-received Cycom 5320-1 prepreg was measured to be 181 J/g. The cured panels were tested at twelve locations across the part to evaluate their DOC, where a false colour image of the data is shown in Figure 5a. This false colour image is a visual representation of the spatial distribution of the state of cure across the composite panel. The discrete DOC points are plotted on the image at the same x-y coordinates as where the DSC samples were extracted from the panel (and alongside temperature information provided by the FOTs). DOC percentage values were calculated using Equation (2). Note that the image corresponds to the configuration of the microwave and laminate as shown in Figure 4b. These tests were conducted to provide an indication of the cure homogeneity of the composite.

From the results in Figure 5a, the average DOC of the composite laminate is >90%, with the exception of two outliers located in similar areas to FOT-A (2.5 mm) and FOT-B (5 mm) and measuring at 78.5% and 85.5%, respectively. When aligning the DOC results with the findings in Figure 4a,b, there is a general correlation in that the thinnest regions (coldest during cure) show the lowest DOC, although it also appears that the longitudinal edges of the panel are better cured than the centre. The results of the degree of cure measurements combined with the thermal camera images and FOT data indicate that there are two main effects combining to result in an inhomogeneous DOC/temperature distribution. Firstly, there is a front/rear gradient with the regions of the panel furthest rearwards exhibiting the highest temperatures and DOC. This can be explained as above given that these are the thickest regions. In addition, there appears to be a secondary effect across the width of the panel, whereby the longitudinal edges experienced the highest temperatures and consequently have the highest DOC, while the centreline of the panel experienced less temperature and displayed a lower DOC. This is likely due to the geometry of the microwave chamber, in that the waveguides are run lengthways along the vertices of the hexagon shaped chamber; thus, the longitudinal edges of the panel are simply closest to the waveguides (see Figure 1). Regardless of the reasons for the inhomogeneity, the area of the panel experiencing the lowest temperatures and DOC was the front central region; therefore, a decision was made to position the MAM there for the subsequent cure to compensate for this cold spot.

#### 3.2.2. Composite Laminate Using a MAM

An identical lamination process was conducted to produce the second composite panel with a 500 mm × 500 mm section of MAM-2 added to the central front region of the panel. FOTs were used to monitor the temperature over the panel, and the temperature-time-power data recorded during the cure cycle is shown in Figure 4c. From these results, FOT-A followed the desired temperature setpoint, FOT-B experienced a temperature gradient of approximately 15 °C, and FOT-C continuously remained below the set temperature by 60–90 °C. The IR image in Figure 4d supports the FOT readings, in that the composite laminate appears to experience heat in the locations of FOT-A and FOT-B, although FOT-C remains cold (10 mm thick composite). This is clearly due to the high level of energy absorption by the MAM in the central front region.

When relating the results from Figure 4c,d to the DSC results of the composite panel in Figure 5b, the temperature distribution seen from both the FOTs and thermal images is supported. The addition of the MAM had a substantial effect on the temperature distribution across the composite and reversed the most and least cured regions of the component. All regions of the panel were under-cured, as the panel did not reach the required temperature for sufficient time. The region under the MAM was least so (DOC values of 82.2% and 76.9%), while the rest of the panel was highly under-cured (<50%). This shows that the MAM was over-effective in absorbing energy, so that while the MAM heated very effectively and followed the setpoint, this did not transmit effectively to the laminate. The PID control of the microwave was set to control from the highest reading, and picked out the readings from the MAM to control this to the setpoint, meaning adequate power was not applied to heat other areas of the laminate sufficiently. Even the laminate directly under the MAM was not sufficiently directly heated and, being separated from the MAM by a layer of thermally insulating breather, did not receive enough conducted heat either. Therefore, it is clear that the cold spot identified in the first trial was far over-compensated for. It should be remembered that the least effective of the two MAMs trialled in the test bed was selected for this trial; however, it is still over-effective in this situation. While it is disappointing that the homogeneity of the heating was lower when the MAM was used, it very clearly demonstrates that MAMs are extremely effective (over-effective in this case) at modifying the temperature distribution. With correct tailoring of MAM properties, it should be possible to reduce their effectiveness to an optimal level to boost heating in the areas of a component that are not so easily heated (regardless of the reason for this lack of heating, whether component geometry or location in the chamber) without over-shooting this goal.

## 4. Conclusions

A barrier to the adoption of microwave processing has been the uneven distribution of heat in a component, where hot and cold spots can appear across a composite part due the interaction of the electromagnetic field within the chamber and the component geometry. As part of this work, two absorbers were utilised to identify their potential to locally enhance the heating effect with the aim of improving the uniformity of the cure of a composite. By conducting dielectric trials at elevated temperatures alongside cure data and thermal diffusivity properties, it was found that a commercially available absorber was able to locally enhance the heating effect greatly. These findings indicate that absorbers have the potential to be used to selectively control the heating of a composite, and therefore have the potential to homogenise both heating and cure across an inhomogeneous component. The control strategy and the location of controlling thermocouples will also have to be carefully chosen to heat the laminate to the required temperature rather than controlling power according to the temperature of the MAM.

## Figures and Tables

**Table 1:** Thermal properties of the microwave absorbing materials.

Microwave Absorbing Materials (MAMs)	Thermal Conductivity/(W/mK)	Thermal Diffusivity/(mm^2^/s)
Silicone	0.36	0.21
MAM-1—Silicon Carbide (35 wt %)/Carbon Black (5 wt %)	0.71	0.46
MAM-2—Aluminium/Carbon Black (Material SFL8925/26 Kraiburg TPE GmbH & Co. KG)	1.39	0.90

## Data Availability

Data can be obtained upon request.
